# An intracochlear electrocochleography dataset - from raw data to objective analysis using deep learning

**DOI:** 10.1038/s41597-023-02055-9

**Published:** 2023-03-22

**Authors:** Klaus Schuerch, Wilhelm Wimmer, Adrian Dalbert, Christian Rummel, Marco Caversaccio, Georgios Mantokoudis, Tom Gawliczek, Stefan Weder

**Affiliations:** 1 Department of ENT, Head and Neck Surgery, Inselspital, Bern University Hospital, University of Bern, Bern, Switzerland; 2 Hearing Research Laboratory, ARTORG Center for Biomedical Engineering Research, University of Bern, Bern, Switzerland; 3 Department of Otorhinolaryngology, Head&Neck Surgery, University Hospital Zurich, University of Zurich, Zurich, Switzerland; 4 Support Center for Advanced Neuroimaging (SCAN), University Institute for Diagnostic and Interventional Neuroradiology, Inselspital, Bern University Hospital, University of Bern, Bern, Switzerland

**Keywords:** Evoked potentials, Diagnostic markers, Diagnostic markers, Translational research, Scientific data

## Abstract

Electrocochleography (ECochG) measures electrophysiological inner ear potentials in response to acoustic stimulation. These potentials reflect the state of the inner ear and provide important information about its residual function. For cochlear implant (CI) recipients, we can measure ECochG signals directly within the cochlea using the implant electrode. We are able to perform these recordings during and at any point after implantation. However, the analysis and interpretation of ECochG signals are not trivial. To assist the scientific community, we provide our intracochlear ECochG data set, which consists of 4,924 signals recorded from 46 ears with a cochlear implant. We collected data either immediately after electrode insertion or postoperatively in subjects with residual acoustic hearing. This data descriptor aims to provide the research community access to our comprehensive electrophysiological data set and algorithms. It includes all steps from raw data acquisition to signal processing and objective analysis using Deep Learning. In addition, we collected subject demographic data, hearing thresholds, subjective loudness levels, impedance telemetry, radiographic findings, and classification of ECochG signals.

## Background & Summary

Electrocochleography (ECochG) measures electrophysiological inner ear potentials in response to acoustic stimulation. These potentials reflect the state of the inner ear and provide important information about its residual function. ECochG is an umbrella term covering four different signal components, i.e., i) the cochlear microphonic (CM, outer hair cell response), ii) the auditory nerve neurophonic (ANN, early neural and inner hair cells response), iii) the compound action potential (CAP, early auditory nerve response), and iv) the summating potential (SP, mainly inner hair cell response)^1–5^.

In cochlear implant (CI) patients, using the implant electrode, we can measure ECochG signals directly within the cochlea. The measurements can be performed during and after implantation. During the implantation process, studies have shown that abrupt signal changes can be caused by traumatic forces^6–14^. Hence, real-time ECochG traces can complement the haptic perception of the surgeon^6,8–11,15–20^. ECochG can also be useful in the post-operative phase, where patients may lose residual cochlear function^21,22^. Most commonly, such losses occur during the first six to twelve months after implant surgery^23–25^ due to different intra-cochlear factors (e.g., immune response to the electrode, intracochlear inflammatory reactions, and intracochlear scar tissue formation)^14,26,27^. However, the underlying mechanisms remain poorly understood and require further research^24^. In summary, in CI recipients, during and after implant surgery, ECochG measurements map cochlear health and thus have great potential to improve our understanding of cochlear function in response to the implant electrode.

The interpretation of ECochG signals, however, is not trivial and requires training. The signal amplitude and signal-to-noise ratio (SNR) can vary greatly among individuals. Furthermore, the morphology and latency of ECochG traces are affected by the remaining neurosensory cells^10,28–30^.

Until recently, the evaluation of ECochG signals was based on visual analysis by experts. This approach has several disadvantages, e.g., a high level of experience is needed, and expert-dependent analysis can lead to a lack of reproducibility, limiting the application of these measurements. We previously introduced a machine learning-based, objective method to determine whether an ECochG signal is present or not^31^. Thereby, three experts labelled more than 4,000 ECochG signals to train and test the machine learning algorithm (consisting of preprocessing steps and a convolutional neural network, CNN).

The aim of this data descriptor is to provide the research community access to our comprehensive electrophysiological data set and algorithms (i.e., raw data with access down to single epoch level, pre-processing and SNR enhancing algorithms, visually labeled data by three independent human experts, and the trained deep learning network AlexNet)^31^. These data are complemented by the measured hearing thresholds, subjective loudness data, demographic data, impedance telemetry measurements, and radiographic parameters.

Potential applications of this data set include, but are not limited to (i) refinement and further use of the deep learning network^31^, (ii) improvement of pre-processing and SNR-enhancing algorithms and data analysis^16,31–33^, (iii) correlation of ECochG signal components and impedance measurements with hearing thresholds^15,16,21,22,34^, (iv) longitudinal evaluation and repeatability assessment of ECochG data^21^, and (v) correlation of multi-frequency and broad-band ECochGs with pure tone ECochGs and hearing thresholds^35^.

## Methods

The data presented in this descriptor were collected in a study that was approved by our local institutional review board (The Cantonal Ethics Committee of Bern, BASEC ID 2019-01578). All participants gave written consent and consent to the use of properly anonymized data before participation.

### Subject demographics

**Fig. 1:**
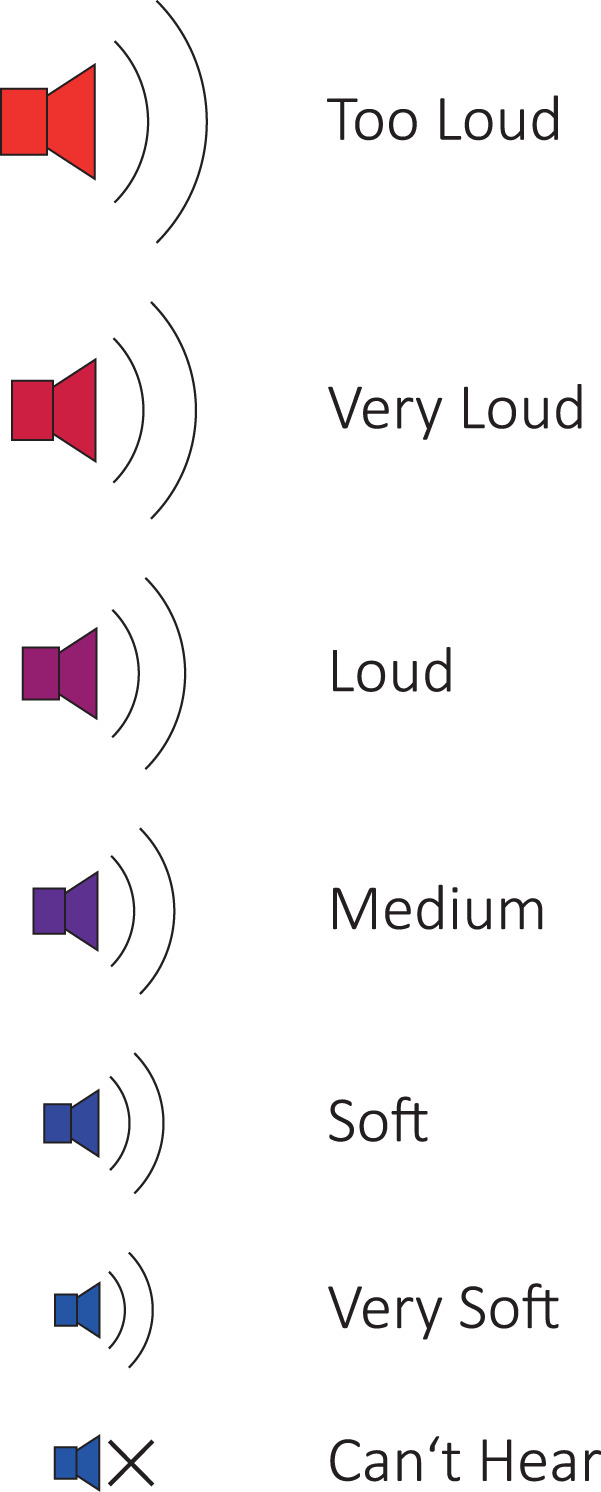
Categories of subjective loudness. Subjects from cohort B classified each acoustic stimulus intensity to one of these categories.

We recorded ECochG traces from 41 adult subjects (n = 46 ears) using a cochlear implant (MED-EL, Innsbruck, Austria). The subjects’ mean age was 58 years (SD = 17.4 yrs, range: 21 to 86 yrs). Pure tone audiograms were performed in a certified acoustic chamber with a clinical audiometer (Interacoustics, Middelfart, Denmark). Hearing thresholds were collected either immediately pre-operatively (cohort A) or, in the case of post-operative measurements (cohort B), on the day of ECochG measurement. We obtained pure tone air conduction hearing thresholds in dB hearing level (HL) at 125, 250, 500, 750, 1000, 1500, 2000, and 4000 Hz. For cohort A, we only included subjects with a hearing threshold at 500 Hz of 100 dB hearing level (HL) or better. For cohort B, we only considered subjects with stable acoustic hearing six months or longer after the implantation. The acoustic hearing was considered stable if the hearing thresholds varied less than 10 dB. In cohort B, subjects categorized the loudness of the acoustic stimulus according to Fig. 1^36^.

### ECochG data

ECochG recordings were performed using MED-EL Maestro research software (versions 8.03 AS and 9.03 AS). The acoustic stimulus was generated by a Dataman 531 waveform generator (Dataman, Maiden Newton, UK) and converted to sound by an Etymotic ER-3C transducer (Etymotic, Grove Village, IL, USA). The acoustic stimulus was triggered via the MED-EL MAX interface. Further details are available in^19^.

**Fig. 2:**
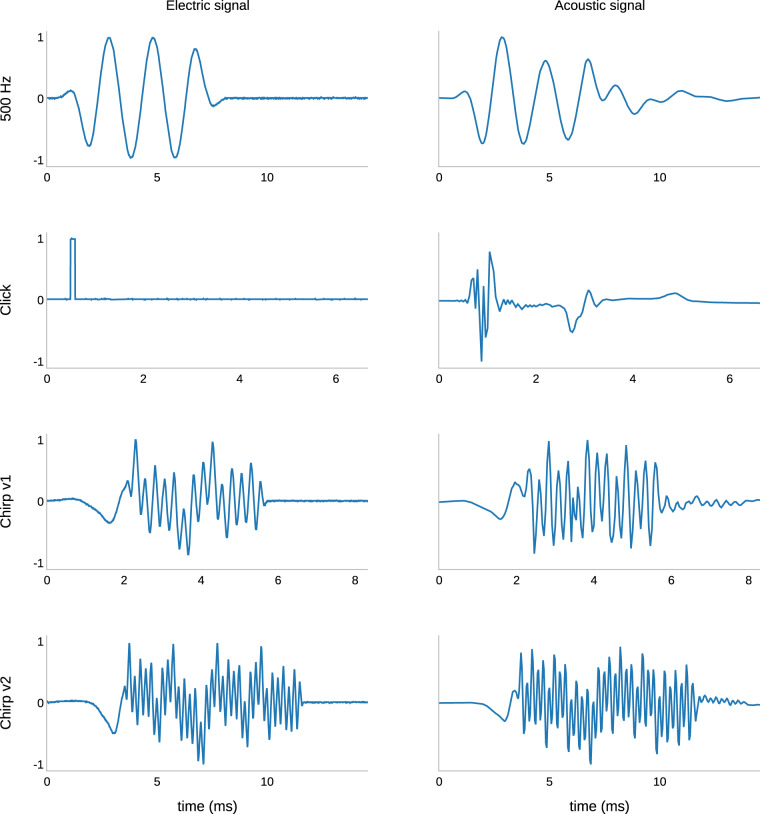
Electrical signals (left) and acoustic signals (right) generated by the waveform generator and transducer, respectively: A) 500 Hz pure tone, B) click, C) SPL chirp v1, and D) SPL chirp v2 stimulus. Note the different time axes (X-axis) scaling. The amplitude axes (Y-axis) were normalized. Acoustic signals were measured using a head and torso simulator (Type 5128-C-111, Brüel & Kjær, Virum, Denmark) and an audio analyzer (XL2, NTi Audio AG, Schaan, Lichtenstein). Electrical signals were measured using an oscilloscope (TDS 1002B, Tektronix, Beaverton OR, USA).

We measured ECochG signals in response to pure tone, click, and SPL chirp stimulus (see Table 1 and Fig. 2). We recorded two polarities (condensation, CON, and rarefaction, RAR) and 100 repetitions (epochs) each. All ECochG recordings were measured in a stable electrode position; either in the operating room after completed electrode insertion (cohort A, 25 ears, the measurement setup can be found in^19,37^) or in a post-operative setting (cohort B, 21 ears) in a certified acoustic chamber. We thereby measured ECochG traces at electrodes 1 (most apical electrode), 4, 7, and 10 and in response to 3 different sound intensity levels (supra-threshold level, near-threshold level, sub-threshold level). The intensity levels were calculated using the individual hearing thresholds measured before the experiment. Our goal was to evoke responses with different SNRs. For cohort B, to obtain longitudinal data, we repeated ECochG recording three times: i) at least 6 months after insertion; ii) within 2 to 48 hours after the first measurement; and iii) 2 to 4 months after the first measurement.

**Table 1:** Table 1 shows the different stimulation modalities with the three stimuli used, the frequencies, the stimulus duration, and the measurement window.

Stimulus type	Frequency (Hz)	Stimulus duration (ms)	Measurement window (ms)
Pure tone	250	12	19.1
Pure tone	500	8	9.6
Pure tone	750	6.67	9.6
Pure tone	1000	5	8.0
Pure tone	1500	4	8.0
Pure tone	2000	3	6.5
Click	NA	0.1	6.5
SPL chirp v1	500	6	12.8
	1000		
	2000		
	4000		
SPL chirp v2	250	12	19.1
	500		
	1000		
	2000		
	4000		

SPL chirp stimuli are superpositions of multiple pure tone frequencies (see Fig. 2).

### Data preprocessing

To pre-process ECochG signals, we implemented the following steps (see^31^ for further details): i) if needed, removal of stitching artifacts; ii) application of a Gaussian-weighted averaging method adapted from^33^ to remove uncorrelated epochs; and iii) application of a second-order Butterworth band-pass filter in forward-backward filtering mode (cutoff frequencies at 10 Hz/5 kHz for visual analysis, and 100 Hz/5 kHz for the objective algorithms). The SNR was calculated using the ± averaging method^38^. The pre-processing steps above were performed using the Python script *do_preprocessing.py*, which is available at^39^.

### Data analysis

For further analysis, we calculated the different ECochG signal components. We highlighted the CM signal by subtracting the CON and RAR responses^40^. Since the subtracted result can also contain other ECochG components, we will refer to the term “CM/DIF” signal in the following text^32^. We calculated the ANN signal by adding the ECochG response to CON and RAR stimulus^3^. For the following text, we will refer to it as “ANN/SUM” response.

**Fig. 3:**
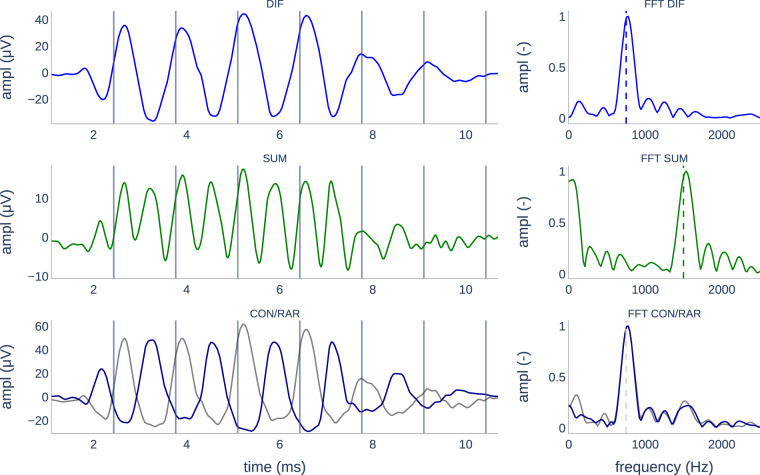
Visual analysis of ECochG traces was performed using six subplots. A) CM/DIF trace, C) ANN/SUM trace, E) CON and RAR traces, and B, D, F) their FFT traces. The gray vertical lines indicate the stimulus period. The dashed vertical lines indicate the expected frequency of the response.

For the visual analysis, the data were labeled by three independent experts with several years of experience in the field. Data were presented using Labelbox^41^ presenting a figure showing i) the CM/DIF trace, ii) the ANN/SUM trace, iii) the CON and RAR traces, and iv-vi) their corresponding Fast Fourier Transform (FFT) magnitude spectra. An example is shown in Fig. 3. During the labeling process, the focus lay on the identification of CM/DIF responses and their binary labeling (ECochG response visible/not visible). Thereby, the experts were forced to make a judgment; otherwise, it was not possible to proceed to the next signal trace. For the labeling of the ANN/SUM and CAP responses, however, in case of ambiguity, the answer could be skipped. The examiners did not discuss their evaluation to avoid bias in the assessment. Signals that were classified as visible CM/DIF responses by two examiners and as noise by the third examiner were presented a second time. Only, if all three experts rated a signal as visible (in the second round), it was marked as such. This was done to avoid volatility errors. Finally, we used the labeled responses to train the deep learning algorithm presented in^31^.

### Impedance telemetry

Before each measurement session, we performed impedance telemetry measurements. We used the default settings of the recordings, recommended by the manufacturer. A charge-balanced, rectangular biphasic cathodic first pulse with a duration of 26.67 μs and an amplitude of 302.4 cu (one current unit, cu, is equivalent to approximately one μA) was used for stimulation resulting in a stimulation charge of 8.06 qu (one charge unit, qu, is equivalent to approximately one nC)^42^. The voltage potential was measured at the end of the anodic phase with respect to the ground electrode located at the implant housing^43,44^.

### Anatomy

Anatomical features were extracted from the Computed tomography (CT) scans using Otoplan (ver. 1.02, CAScination, Bern, Switzerland)^45^. CT images with a slice thickness equal to or less than 0.3 mm were used. Markers to define the cochlea were set (A value, distance between the round window and the contralateral wall of the cochlea, B value, width of the cochlea perpendicular to the A value, H value, distance from the basal turn to the apical center)^46,47^.

## Data Records

**Fig. 4:**
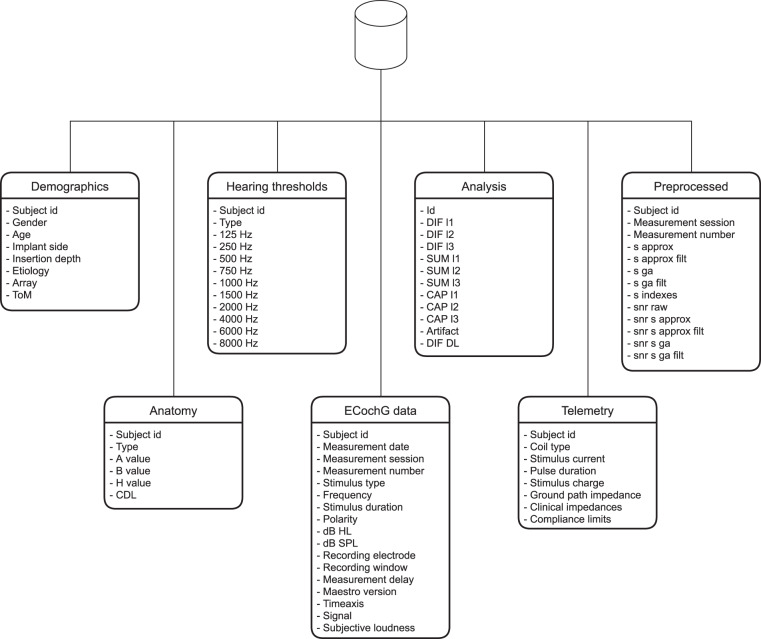
The Bern ECochG database contains seven tables.

All data created during this research project are accessible from the Dryad repository^39^. The dataset is stored in the Bern ECochG SQL database, and consists of seven tables, as shown in Fig. 4. Each table can be accessed individually. All tables except the *Analysis* table use the common *Subject id* attribute, which can be employed to connect the tables.

### Subject demographics

The subject’s demographic data is stored in the *Demographics* table. A list of all attributes is available in Table 2. The *Subject id* is stored as XX_Y, where XX is post-insertion (PI) or post-operative (PO) and Y is an incrementing number for each subject. The Python script *demographics.py* illustrates how to access the demographic data.

**Table 2:** Structure of the subject *Demographics* table.

Attribute	Data type	Description
Subject id	String	PI: post-insertion, PO: post-operative
Gender	String	F: female, M: male
Age	Int	Subject age (years)
Implant side	String	L: left, R: right
Insertion depth	Int	Number of electrodes inside the cochlea
Etiology	String	Cause of hearing loss
Array	String	Implant electrode array type
ToM	Int	First recording time after surgery (months)

### Hearing thresholds

The subjects’ hearing thresholds are stored in table *Hearing thresholds*. A list of all attributes can be found in Table 3. For cohort A, we provide immediate, pre-operative and 3–5 weeks post-operative hearing thresholds. For cohort B, we list the hearing threshold before the first post-operative ECochG recording (post-operative) and before the third post-operative recording (post-operative 2). In case of a missing hearing threshold, we left cells blank.

**Table 3:** Structure of the *Hearing thresholds* table.

Attribute	Data type	Description
Subject id	String	PI: post-insertion, PO: post-operative
Type	String	pre-operative, first post-operative, post-operative, post-operative 2
125 Hz	Int	
250 Hz	Int	
500 Hz	Int	
750 Hz	Int	
1000 Hz	Int	
1500 Hz	Int	
2000 Hz	Int	
3000 Hz	Int	
4000 Hz	Int	
6000 Hz	Int	
8000 Hz	Int	
Click	Int	
SPL chirp v1	Int	
SPL chirp v2	Int	

Hearing thresholds were measured in dB hearing level (HL).

### ECochG data

The table *ECochG* contains all ECochG raw data. A list of all attributes can be found in Table 4. The *measurement date* shows when the measurement was performed. *Measurement session* indicates to which session the measurement belongs (0: post-insertion, cohort A, 1–3: post-operative measurements, cohort B). *Measurement number* is an ascending number for each session. *stimulus type* indicates which acoustic stimulus was used for the recording. *Stimulus duration* indicates the duration of the acoustic stimulus in milliseconds (ms). *Polarity* indicates whether a CON or RAR stimulus was used. The acoustic amplitude of the stimulus is given in dB hearing level (*dB HL*) for pure tones or in dB peak equivalent sound pressure level *dB p.e. SPL* for click and SPL chirp stimulus^29^. The *Recording window* indicates the length of the recording in ms. The *Measurement delay* specifies the delay between the start of the acoustic stimulus and the start of the measurement window. In most cases, *Measurement delay* is set to 1 ms. *Timeaxis* and *Signal* are Numpy arrays stored as JSON strings^48,49^. The *Timeaxis* was stored as a 1 × N array, where N indicates the time samples. The *Signal* was stored as M × N, where M indicates the recorded epochs and N indicates the recording samples. *Subjective loudness* represents the loudness of the acoustic stimulus as perceived by the subjects (cohort B). Available responses are shown in Fig. 1.

**Table 4:** Structure of the *ECochG* table.

Attribute	Data type	Description
Subject id	String	PI: post-insertion, PO: post-operative
Measurement date	String	YYYY-MM-DD
Measurement session	Int	0: post-insertion, 1–3: post-operative
Measurement number	Int	Incrementing number
Stimulus type	String	Pure tone, Click, SPL chirp v1, SPL chirp v2
Frequency	Int	In Hz; 0 for non-pure tones
Stimulus duration	Float	ms
Polarity	String	CON, RAR
dB HL	Int	Stimulus intensity (dB HL)
dB SPL	Int	Stimulus intensity (dB SPL)
Recording electrode	Int	
Recording window	Float	Recording window duration (ms)
Measurement delay	Float	Recording delay (ms)
Maestro version	String	Software version used for recording
Timeaxis	Array	Timeaxis of the recording
Signal	Array	Recorded epochs
Subjective loudness	String	Subjective perception of the stimulus

### Preprocessed

The *Preprocessed* table contains data generated after the pre-processing steps. The attributes are listed in Table 5. The signal is indicated by *s*.

### Analysis

The *Analysis* table contains the visual and objective analysis of the signals. The analyzed signals consist of a pair of CON and RAR recordings. The recordings can be traced using the *Id*, which is represented as XX_Y.SESSION_NR.NR_CON.NR_RAR. Where XX is PO or PI, Y represents the subject’s incrementing identification number, SESSION_NR is the session number, and NR_CON and NR_RAR represent the measurement numbers (e.g., PO_1.1.010.011 consists of recordings #10 and #11 of post-operative subject 1 and session 1, respectively).

**Table 5:** Structure of the *Preprocessed* table.

Attribute	Data type	Description
Subject id	String	PI: post-insertion, PO: post-operative
Measurement session	Int	0: post-insertion, 1–3 post-operative
Measurement number	Int	Incrementing number
s approx	Array	Mean of all epochs
s approx filt	Array	Bandpass filtered mean of all epochs
s ga	Array	Mean of correlated epochs
s ga filt	Array	Bandpass filtered mean of correlated epochs
s indexes	Array	Indexes of correlated epochs
snr raw	Float	SNR of raw signal
snr s approx	Float	
snr s approx filt	Float	
snr s ga	Float	
snr s ga filt	Float	

Analysis was performed for CM/DIF (*DIF*), ANN/SUM (*SUM*), and *CAP* components. ECochG components were labeled by the examiners (*l1* - *l3*) and the deep learning (*DL*) algorithm.

Objective analysis of the CM/DIF signals is only available for pure tone stimulus. Unlabeled components were left blank. Table 6 shows an overview of all attributes available in the *Analysis* table.

**Table 6:** Structure of the *Analysis* table.

Attribute	Data type	Description
Id	String	XX_YY.SESSION_NR.nr_CON.nr_RAR
DIF l1	Bool	examiner 1
DIF l2	Bool	examiner 2
DIF l3	Bool	examiner 3
SUM l1	Bool	
SUM l2	Bool	
SUM l3	Bool	
CAP l1	Bool	
CAP l2	Bool	
CAP l3	Bool	
Artifact	Bool	Artifact present yes (1)/no (0)
DIF DL	Bool	Deep learning

### Anatomy

The *Anatomy* table contains the anatomical features. A list of all attributes can be found in Table 7. *Type* indicates whether the anatomical features were extracted from pre-operative or post-operative CT images. The shape of the cochlea is indicated by the *A*, *B*, and *C* values, and the cochlear duct length (*CDL*)^46^. General statistics about the anatomical features are shown in Table 8

**Table 7:** Structure of the *Anatomy* table.

Attribute	Data type	Description
Subject id	String	PI: post-insertion, PO: post-operative
Type	String	pre-operative, post-operative
A value	Float	
B value	Float	
H value	Float	
CDL	Float	Cochlear duct length

**Table 8:** General statistics about the anatomical features extracted from CT scans.

	A value (mm)	H value (mm)	B value (mm)	CDL (mm)
Mean	9.13	3.30	7.09	36.92
SD	0.60	0.38	0.59	2.53
Max	10.6	4.6	8.3	42.1
Min	8	2.3	6	33

### Impedance telemetry

The table *Telemetry* contains the recorded values during clinical routine telemetry measurements. A list of all attributes can be found in Table 9. The *Clinical impedances* represent the impedances from the electrodes (1 to 12) to the ground electrode. General statistics about clinical impedances are shown in Table 10

**Table 9:** Structure of the *Telemetry* table.

Attribute	Data type	Description
Subject id	String	
Coil type	String	
Stimulus current	Float	current unit (cu)
Pulse duration	Float	millisecond (ms)
Stimulus charge	Float	charge unit (qu)
Ground path impedance	Float	Resistance (Ω)
Clinical impedances	Array	Resistance (Ω); electrodes 1 to 12
Compliance limits	Array

**Table 10:** General statistical data on impedance measurements with electrode one as the most apical.

	El. 1	El. 2	El. 3	El. 4	El. 5	El. 6	El. 7	El. 8	El. 9	El. 10	El. 11	El. 12	All
Median	5000	4370	4270	3725	3900	3155	3540	3635	4230	4210	4030	4185	3990
Mean	5908	4955	4543	4045	4001	3415	3665	3948	4218	4704	4532	5002	4411
SD	2872	2785	1523	1175	12267	798	948	1423	1448	2860	2422	2648	2102
Max	20300	21130	8200	7400	7360	5320	6460	8980	8840	20400	15460	14460	20300
Min	3170	2420	2600	2420	2420	2400	2400	2240	2140	2330	1610	1940	1610

## Technical validation

The ECochG system was calibrated by the manufacturer. No changes were made to the recorded raw data. To increase the reliability of the measurements in cohort A, we used sterile eartips for recording and applied the guidelines presented in^37^. In cohort B, we compared hearing thresholds measured with the ECochG hardware with the audiogram before each measurement session. In this way, we were able to verify that the eartips were placed correctly. For this purpose, we used the customized software *AcousticStimulatorGUI*, available from^39^. This software interacts directly with the Dataman waveform generator and allows the use of customized acoustic stimulus. The software with the corresponding hardware was calibrated on a head and torso simulator (Brüel & Kjær, type 5128, Nærum, Denmark). The *AcousticStimulatorGUI* was calibrated with our hardware. Using this software together with other hardware requires a new calibration. The calibration parameters can be adjusted in the *GetFrequencyOffset* method of the *Dataman* class.

## Usage Notes

The database has been split into seven data parts and the empty Bern_ECochG database to facilitate downloading. Each part is saved as a .sql file and can be imported into the Bern_ECochG database individually. We recommend downloading all parts and assembling them using *sqlitebrowser* available at https://sqlitebrowser.org/. The Python scripts provided will only work when the database is fully assembled. The Python scripts show how to access the database. Along the Python scripts, a .yml file is provided to install all dependencies to run the scripts.

## Data Availability

The code used to create and process the presented data is provided in^39^ or is part of open-source repositories^48–53^.
